# Wearable Sensors and Systems in the IoT

**DOI:** 10.3390/s21237880

**Published:** 2021-11-26

**Authors:** Subhas Chandra Mukhopadhyay, Nagender Kumar Suryadevara, Anindya Nag

**Affiliations:** 1 School of Engineering, Macquarie University, Sydney, NSW 2109, Australia; 2 School of Computer and Information Sciences, University of Hyderabad, Hyderabad 500046, Telangana, India; nks@uohyd.ac.in; 3 Faculty of Electrical and Computer Engineering, Technische Universität Dresden, 01062 Dresden, Germany; anindya.nag@tu-dresden.de; 4 Centre for Tactile Internet with Human-in-the-Loop (CeTI), Technische Universität Dresden, 01069 Dresden, Germany

Wearable smart devices are widely used to determine various physico-mechanical parameters at chosen intervals. The proliferation of such devices has been driven by the acceptance of enhanced technology in society. Despite the exponential growth of wearable sensors, there are limitations in terms of the broader aspects of their commercialized uses, which need improvement to further enhance the field of wearable electronics.

The Internet of Things (IoT) is responsible for connecting smart objects around the world and has led to the proliferation of these smart objects over the last two decades. This Special Issue aims to present the issues and challenges faced by the currently proposed IoT-based systems in addition to state-of-the-art research on the commercialization of the current systems.

This editorial summarizes the published papers in this Special Issue on Wearable Sensors and Systems on the Internet of Things theme of applications.

The global market for Internet of things (IoT) technology reached USD 100 billion for the first time in 2017, with a forecast to further grow to around USD 1.6 trillion by 2025, according to [1]. The global internet of things in the healthcare market was valued at USD 113.75 billion in 2019 and is expected to reach USD 332.67 billion by 2027, registering a compound annual growth-rate of 13.20% from 2020 to 2027 according to [2].

Human-activity monitoring is a vibrant area of research with the potential for commercial development, which is discussed by Mukhopadhyay [3]. It is expected that many more lightweight, robust, and efficient wearable devices will be available for monitoring a wide range of activities. The development of lightweight physiological sensors will lead to the formation of comfortable wearable systems to monitor different ranges of inhabitant-related activities. Formal and informal surveys predict an increase in the interest in, and consequently usage of, wearable devices in the near future, where the cost of such devices is expected to fall with an increased use.

Wearable devices have been a state-of-the-art technology and associated with IoT for quite some time. The full range of new capabilities of these IoT-based wearable sensors has been realized every day for their pervasive connectivity. Traditional remote healthcare information systems involve data transfer, signal processing mechanisms, and naive machine learning models deployed on remote servers to process the medical data of patients. Some of the shortcomings of this technique include an unsuitability for resource-constrained wearable IoT devices. Resources such as processing, memory, energy, and networking capability are limited in wearable IoT (WIoT) devices.

The lack of optimization for resource usage, prediction of medical conditions, and dynamic assessment on the basis of available information related to wearable systems has led to several innovations in the context of IoT devices and applications.

Wearable devices play an important role in terms of the measurement and collection of data. Using the IoT gateway as an intermediate hub between the wearable devices and the IoT server, bidirectional communication between the end user and medics can be established in real time Haghi et al. [4].

A patient-specific (PS) fall prediction and detection prototype system was introduced by Sadeh et al. [5]. The system utilizes a single tri-axial accelerometer attached to the patient’s thigh to distinguish between activities of daily living (ADL) and fall events. The proposed system consists of two modes of operation, including fast and slow modes. The fast mode for fall predication (FMFP) predicts a fall event (300–700 msec) before it occurs. The accuracy of the proposed algorithms are further validated via the MobiFall Dataset. The FMFP achieves a sensitivity and specificity of 97.8% and 99.1%, respectively, while the slow mode for fall prediction (SMFD) achieves a sensitivity and specificity of 98.6% and 99.3%, respectively, for a total number of 600 measured falls and ADL cases from 77 subjects.

Prabhu et al. [6] stated that generally minor or no symptoms of kidney diseases were seen until a significant loss of kidney function was observed and/or a disease state related to kidney malfunction worsened. Creatinine and Blood Urea Nitrogen (BUN) are considered biomarkers for kidney healthcare. The authors have demonstrated the development of an IoT-based point-of-care (PoC) diagnostic device protocol for the prognostic and prophylactic management of creatinine from serum. The biosensing device has the potential to detect the creatinine from serum samples in an acceptable range, with a maximum concentration of 50 ppm. This highest limit is over three times higher than the acceptable range. The sensor was calibrated with standard laboratory procedures, but the performance and accuracy level were not tested with a pathological estimation by using real samples due to human ethics issues.

Malhi et al. [7] have presented applied research in order to monitor physiological parameters, such as skin temperature, heart rate, and body impact. The novel aspect of their design is the low cost and ease of detection of medical distress, which does not necessitate pressing any panic button. This is an enormous improvement from existing commercial products. The design of the IR sensors could be improved in order to subsequently decrease their susceptibility to noise, to a point where they could be moved onto the wrist. This would provide a much more comfortable and less intrusive unit, with no need for a finger glove. The monitoring of athletes while exercising would be possible if the sensitivity to movement was decreased.

Suryadevara et al. [8] presented a smart, wireless, and noninvasive sensing system to estimate the amount of fluid loss that a person experiences while performing a physical activity. The system measures three external body parameters—heart rate, Galvanic Skin Response (GSR), and skin conductance—and the skin temperature. Sensors continuously monitor the heart rate, skin conductance, and skin temperature. The amplified and filtered signals are processed by a microcontroller and transmitted wirelessly using ZigBee technology. Quazi et al. [9] developed an algorithm for the automatic recognition of emotions using various clustering techniques. 

An IoT-based portable sensing device was prototyped by Nag et al. [10] to measure the serum concentration of CTx-1 molecules. The developed PoC device was able to measure the concentration of CTx-1 molecules. The developed device can be attached to a toilet for the automatic sampling of urine. The measured data was sent to an IoT-based cloud server. After developing the calibration curve using known-concentration samples, four unknown concentrations of serum samples were tested using the proposed device and were then quantitatively compared with the ELISA-based measurement.

Traditional screening for COVID-19 typically includes survey questions about symptoms and travel history. The authors Quer et al. [11] explored whether personal sensor data collected over time may help identify subtle changes indicating an infection. The authors have developed a smartphone application that collects smartwatch and activity tracker data, as well as self-reported symptoms and diagnostic testing results.

Security is one of the pressing issues of the wider deployment of wearable IoT systems. Pal et al. [12] have identified and examined the state-of-the-art security requirements in IoT. The authors proposed a set of security requirements for an IoT security architecture that could be useful for wearable systems in IoT applications.

Guanglou et al. [13] proposed a finger-to-heart (F2H) IMD authentication scheme to address this trade-off between security and accessibility. An improved minutia-cylinder-code-based fingerprint authentication algorithm was proposed for IMD by reducing the length of each feature vector and the number of query feature vectors. The experimental results showed that the improved fingerprint authentication algorithm significantly reduced both the size of messages in transmission and computational overheads in the device, which could be utilized to secure the IMD.

Many devices and solutions for remote electrocardiogram (ECG) monitoring have been proposed in the literature. These solutions typically have a large marginal cost per added sensor. The authors Spanò et al. [14] proposed an ECG remote monitoring system that is dedicated to nontechnical users in need of long-term health monitoring.

The authors Anindya Nag et al. [10] presented the design, fabrication, and implementation of novel graphite/PDMS sensors for biomedical applications. The sensor patches were employed for strain-sensing purposes by attaching them on different parts of the body such as fingers, elbows, the neck, and knees. Strain sensing was successfully done based on the bending of the different joints on which the sensor patches were attached. The promising results shown by the sensor patches increased the chances of utilizing them in the future in the biomedical world.

Although the research and development on wearable devices has reached a stage where they can be used as normal household items, the high cost is still an impediment. The prices of the products need to decrease to a level that more people can afford. There is a huge market of aging people in Asian countries. The research and scientific communities are working hard to design and develop smart wearable devices to be used for the continuous monitoring of different human activities twenty-four hours a day and seven days a week. The issues of privacy, power consumption, reliable data collection, and patient identification pose challenges for the development of wearable sensing systems for continuous activity monitoring (Mukhopadhyay [3]). 

An overview of sensing platforms in either a wearable form or integrated into the environment published in this Special Issue is presented in the next section.

The paper titled “Medication Adherence and Liquid Level Tracking System for Healthcare Provider Feedback”, authored by Payne et al. [15], developed a sensing system that was capable of detecting eye drop use, measuring the fluid level, and sending information to a healthcare team to facilitate intervention. The study was carried out by utilizing a selected group of off-the-shelf and custom-built sensors in combination with a Bluetooth-enabled device. Tracking a patient’s adherence to medication is an issue that has not been well addressed, especially when it comes to eye drop-based medications. The authors have developed a customized 3D-printed sleeve that fits around a prescription eye dropper. They demonstrated that the system could successfully identify when eye drops were used 97% of the time. The sensors also had a capacitive approach in terms of their applications.

The paper titled “The Wristwatch-Based Wireless Sensor Platform for IoT Health Monitoring Applications”, authored by Kumar et al. [16], focused on the development of an 868 MHz radio and antenna and a gateway to establish wireless communication between a wristwatch sensor platform and a smartphone application. The design and development of a novel wristwatch-based wireless sensor band was completed; an example application demonstrating the sensor platform operated at the sub-GHz (868 MHz) ISM band. The sensor device, incorporating an SpO_2_ and a heart rate sensor, communicated with a gateway using an 868 MHz Mi-Wi network protocol. The measurements of the patients were done clinically, where the Mi-Wi wireless network protocol was implemented due to its low data rate, low power, and low complexity. The measured results demonstrated a communication range of approximately 31 m for the 868 MHz sensor platform, which was approximately four times greater than for the commercially available Fitbit Charge 3 BLE wristwatch devices.

The paper titled “A Flexible Strain Sensor Based on the Porous Structure of a Carbon Black/Carbon Nanotube Conducting Network for Human Motion Detection”, authored by Zhang et al. [17], developed flexible resistance strain sensors with porous structures. The prototypes were composed of carbon black and multi-walled carbon nanotubes. The advantages of the synergetic effects of mixed carbon black and carbon nanotubes and their porosity show a promising potential for applications in monitoring human motions and physiological parameters.

The paper titled “Automatic Classification of Squat Posture Using Inertial Sensors: Deep Learning Approach”, authored by Lee et al. [18], demonstrated the classified performance of deep learning and compared it to conventional machine learning models for the Inertial Measurement Units (IMUs). While the IMUs were attached to the left thigh, right thigh, left calf, right calf, and lumbar region, the data collected was analyzed using conventional machine learning and deep learning models. The best results were for the right thigh, showing an accuracy of 58.7% for conventional machine learning models.

With the rapid progress of Internet of Things (IoT) techniques, location is a critical information for many fields, and location-based services (LBSs) are widespread and prevalent in people’s daily lives. The paper titled “Cost-Effective Wearable Indoor Localization and Motion Analysis via the Integration of UWB and IMU”, authored by Zhang et al. [19], proposed hardware and data fusion solutions that were capable of motion tracking and localizing the UWB and IMU. Their proposed hardware was robust and was capable of NLoS localization and motion tracking. A compact-sized and energy-efficient wearable indoor localization module with the corresponding algorithms was presented in the paper.

The “Hydrophobic Paper-Based SERS Sensor Using Gold Nanoparticles Arranged on Graphene Oxide Flakes”, authored by Lee and Kim [20], demonstrated the development of AuNPs@GO/h-paper-based SERS sensors, which can be highlighted as a simple, convenient, time-saving, and economical method for the hydrophobic surface modification of filter paper. The hydrophobic treatment increased the contact angle and decreased the contact area of the aqueous solution. The limit of detection was 10 nM with an R^2^ value of 0.966. To demonstrate the possibility of a practical utilization, the presented SERS sensor was used to detect a thiram at the micromolar level to validate the functionality of these sensors at real-time levels. The authors implied that the proposed AuNPs@GO/h-paper-based SERS sensor could be applied for point-of-care diagnostics in daily life and in spacecraft applications.

The epidemic of lifestyle diseases, including diabetes, heart disease, arteriosclerosis, and stroke, is regarded as an urgent issue in the world. The paper titled “WaistonBelt X: A Belt-Type Wearable Device with Sensing and Intervention Toward Health Behavior Change”, authored by Nakamura et al. [21], focus on a device that can automatically measure a waistline with a magnetometer that detects the movements of a blade installed in the buckle in order to monitor the basic activities of daily living with inertial sensors. Furthermore, WaistonBelt X alerts the user about correcting improper lifestyle habits by using a built-in vibration function.

The paper titled “Wellness Assessment of Alzheimer’s Patients in an Instrumented Health-Care Facility”, authored by Masciadri et al. [22], presented a new ICT methodology for wellness assessment to medical personnel to support their activities. The proposed system is constituted by well-being indicators. The first contribution of this paper was the implementation of a coarse-grained low-cost localization system for the continuous monitoring of the position of people wearing Bluetooth bracelets. It pays particular attention to reliability issues and defines a methodology for antennas and their domain knowledge.

The paper titled “A Wide-Range, Wireless Wearable Inertial Motion Sensing System for Capturing Fast Athletic Biomechanics in Overhead Pitching”, authored by Lapinski et al. [23], is a pilot study with simultaneous data collected from an optical 3D motion capture system in the Sports Performance Laboratory and a multi-segment inertial system on two collegiate baseball pitchers.

The paper titled “Multifunctional Flexible Sensor Based on Laser-Induced Graphene”, authored by Han et al. [24], presented the implementation of laser-induced graphene for realizing a flexible sensor for multiple sensing applications. The objective was to improve the electrochemical sensing sensitivity of the sensor, where the effectiveness was validated through the introduction of various interfering ions in the solution, as in the case of real chemical-sensing applications. The effect of bipolar surfactant molecules was also presented in order to improve the out-of-plane resistance of the graphene network and the GF of the sensor.

The paper titled “Zirconia-Based Ultra-Thin Compact Flexible CPW-Fed Slot Antenna for IoT”, authored by Gómez et al. [25], presented an ultra-thin compact flexible CPW-fed slot monopole antenna based on ENrG’s Thin E-Strate, suitable for IoT applications for frequencies of 2.75 GHz and 5.8 GHz. This novel ceramic dielectric—for which the electromagnetic characterization was analyzed—provided 25-fold thinner antennas as compared to Arlon 25N and flexible PP, respectively, which resulted in a size reduction of more than 15%. Antenna prototypes on ENrG’s Thin E-Strate were fabricated using two metallization techniques, including electro-textile and inkjet printing, and have been subsequently characterized in terms of return losses with proper results.

The paper titled “Cancelable Iris- and Steganography-Based User Authentication System for the Internet of Things”, authored by Yang et al. [26], presents a design for a user authentication system for IoT networks. It is equipped with a cancelable iris and a steganography-based mechanism for hiding keys. The feature quantization and shifting processes were conducted using original feature vectors before a random projection-based feature transformation to achieve an optimal recognition performance. The authors proposed to enhance the security of the cancelable iris biometrics using steganography by hiding user-specific keys to address key exposure-related attacks, e.g., ARM, which existing key-dependent cancelable bio-metric systems are susceptible to. The authors explored different types of transformation functions and studies for hiding the secret key under various scenarios, e.g., in a mobile environment.

The paper titled “Evaluation on Context Recognition Using Temperature Sensors in the Nostrils”, authored by Kodama et al. [27], proposed a context recognition method using temperature sensors in the nostrils. The authors selected the optimal sensor suitable for detecting breath from the options of a photo-reflector, humidity sensor, and temperature sensor. The authors confirmed that a temperature sensor was most suitable for the accurate detection of breath. The authors deployed a prototype using temperature sensors and evaluated the recognition of breath, workload, and behavior. The proposed system was able to detect breath, while the temperature behaved differently than that of the baseline flow data. The authors confirmed that the change in the temperature data caused by the workload was different between subjects and did not confirm that nasal congestion changed with respect to the workload. The proposed system could recognize six behaviors, including drinking, resting, eating, walking, laughing, and vocalizing, at an average accuracy of 54%, and eight behaviors, including drinking, resting, eating, walking, laughing, vocalizing, yawning, and sneezing, at an average accuracy of 86%.

The review paper titled “Recent Advances in Fabrication Methods for Flexible Antennas in Wearable Devices: State of the Art”, authored by Mohamadzade et al. [28], checked the possibility of applying 3D direct-write printing to form antenna-ICs. The current advanced manufacturing methods for wearable antennas include the categories of fabric-based embroidered antennas, polymer-embedded antennas, microfluidic antennas with injection alloys, and inkjet screen and 3D-printed antennas. Each of these methods was discussed in detail, in addition to an analysis done on the fabrication process, processed materials, and the antenna’s properties.

This editorial has reviewed the literature from this Special Issue on wearable sensors and devices for monitoring various human-related activities. Human activity monitoring is a vibrant area of research, and significant developments have been reported. It is expected that many more lightweight and high-performance wearable devices will be available for monitoring a wide range of activities. The challenges faced by the current designs will also be addressed in devices developed in the near future. Formal and informal surveys predict an increase in the interest in, and consequently usage of, wearable devices in the near future. The cost of the devices is also expected to fall, resulting in a more widespread acceptance in society. We thank all the authors for sharing their valuable contributions to this Special Issue.
